# Systemic Lupus Erythematosus Developing in a Young African Female Patient With Kikuchi-Fujimoto Disease

**DOI:** 10.7759/cureus.88381

**Published:** 2025-07-20

**Authors:** Kamal Nijim, Karolien Dockerwolcke, Amin Da'meh, Pir Abdul Ahad Aziz Qureshi, Vikram Rao Bollineni

**Affiliations:** 1 Department of Radiology, University Hospital Brussels, Brussels, BEL; 2 Department of Pathology, University Hospital Brussels, Brussels, BEL; 3 Faculty of Medicine, University of Iceland, Reykjavik, ISL; 4 Department of Radiology, Landspítali - University Hospital, Reykjavik, ISL

**Keywords:** autoimmune disease, kikuchi-fujimoto disease, kikuchi-fujimoto disease and systemic lupus erythematosus, kikuchi-fujimoto disease (kfd), systemic lupus erythematosus, viral infection, viral lymphadenopathy

## Abstract

Kikuchi-Fujimoto disease (KFD) is an unusual, benign, and self-resolving etiology of cervical lymphadenopathy. The cause of KFD is still unknown, but it is believed to be triggered by viral or autoimmune mechanisms. It usually has an excellent prognosis and primarily affects Asian women under 30. Although rare, KFD should be considered in patients who present with persistent lymphadenopathy. Diagnosis is based on the histological examination of lymph nodes, which typically reveals necrosis surrounded by histiocytes with crescent-shaped nuclei, immunoblasts, and an absence of neutrophils. Notably, systemic lupus erythematosus (SLE) has been reported to precede, coincide with, or follow a diagnosis of KFD. Here, we present a 24-year-old African woman initially diagnosed with KFD who subsequently fulfilled the diagnostic criteria for SLE. This case report highlights the clinical and diagnostic difficulties presented by this uncommon association, underscoring the need for attentiveness in patients exhibiting persistent lymphadenopathy and possible autoimmune characteristics.

## Introduction

Kikuchi-Fujimoto disease (KFD), also known as histiocytic necrotizing lymphadenitis, is an infrequent and self-limiting condition first described in 1972 by Japanese pathologists Kikuchi and Fujimoto [1,2]. It is primarily seen in young women, particularly those of Southeast Asian descent, and is associated with an overall good prognosis, with most cases resolving spontaneously within one to four months [3]. However, several studies and case series have also reported that lymphadenopathy can persist for several months, even in the absence of specific treatment. For example, Kucukardali et al. reported in their analysis of 244 cases that lymphadenopathy resolved completely or improved within six months [2].

The etiology of KFD remains undefined, but viral and autoimmune triggers may play a significant role, particularly given its association with systemic lupus erythematosus (SLE) [2]. Clinically, KFD often presents with fever, leukopenia, and unilateral cervical lymphadenopathy, most commonly involving the posterior cervical lymph node [1]. Due to its nonspecific presentation, KFD can mimic conditions such as tuberculous lymphadenitis, malignant lymphoma, and other autoimmune or infectious disorders, posing significant diagnostic challenges [2].

Clinicopathological correlation is necessary for diagnosing KFD. Histology is nonspecific but can be suggestive [4]. An excisional biopsy of the affected lymph nodes typically shows necrosis encircled by histiocytes with crescent-shaped nuclei, immunoblasts, and an absence of neutrophils [5]. Radiological findings, while less specific, may show multiple homogeneously enhanced lymph nodes without gross necrosis [6].

KFD is frequently associated with autoimmune diseases such as Sjögren’s syndrome, rheumatoid arthritis, and Still’s disease [4]. Its link with SLE is particularly significant, as KFD may precede, coincide with, or develop after the diagnosis of SLE [3]. Occasionally, KFD presents as a form fruste or early manifestation of SLE, and distinguishing between KFD and SLE may be challenging, particularly in atypical cases. According to Baenas et al., KFD precedes SLE in 30% of cases, occurs simultaneously in 47%, and follows SLE in 23% [1]. Owing to their distinct clinical courses and therapeutic approaches, differentiation between KFD and SLE is essential [7].

## Case presentation

A 24-year-old woman from Guinea presented with left preauricular and bilateral submandibular region swelling accompanied by a high-grade fever (39 °C). On physical examination, bilateral cervical lymphadenopathy was observed, more prominent on the left side. On palpation, notable swelling in the left submandibular area was tender. Diffuse, itchy skin plaques were observed on the face, arms, knees, and other body parts, accompanied by mild facial swelling. The otoscopy results were normal. Laboratory investigations were performed, including a complete blood count, which revealed significant microcytic anemia without evidence of iron deficiency. Hemoglobin electrophoresis was normal, and the Mantoux tuberculin skin test was negative for tuberculosis. Blood investigations were ordered, and the results are outlined in Table 1.

**Table 1:** Blood investigation results of the patient at the time of admission TLC: total leukocyte count; Hg: hemoglobin; MCV: mean corpuscular volume; MCH: mean corpuscular hemoglobin; CRP: C-reactive protein

Investigations	Values on admission	Reference range
TLC	11.3 x 10^9^/L	4.3-9.5 x 10^9^/L
Hg	8.6 g/dL	11.7-15.1 g/dL
MCV	67.5 fL	83.9-98.0 fL
MCH	21.4 pg	27.7-32.8 pg
Platelets	253 x 10^9^/L	150-400 x 10^9^/L
CRP	47.3 mg/L	<5 mg/L
Blood glucose	5 mmol/L	4-6 mmol/L

**Figure 1:**
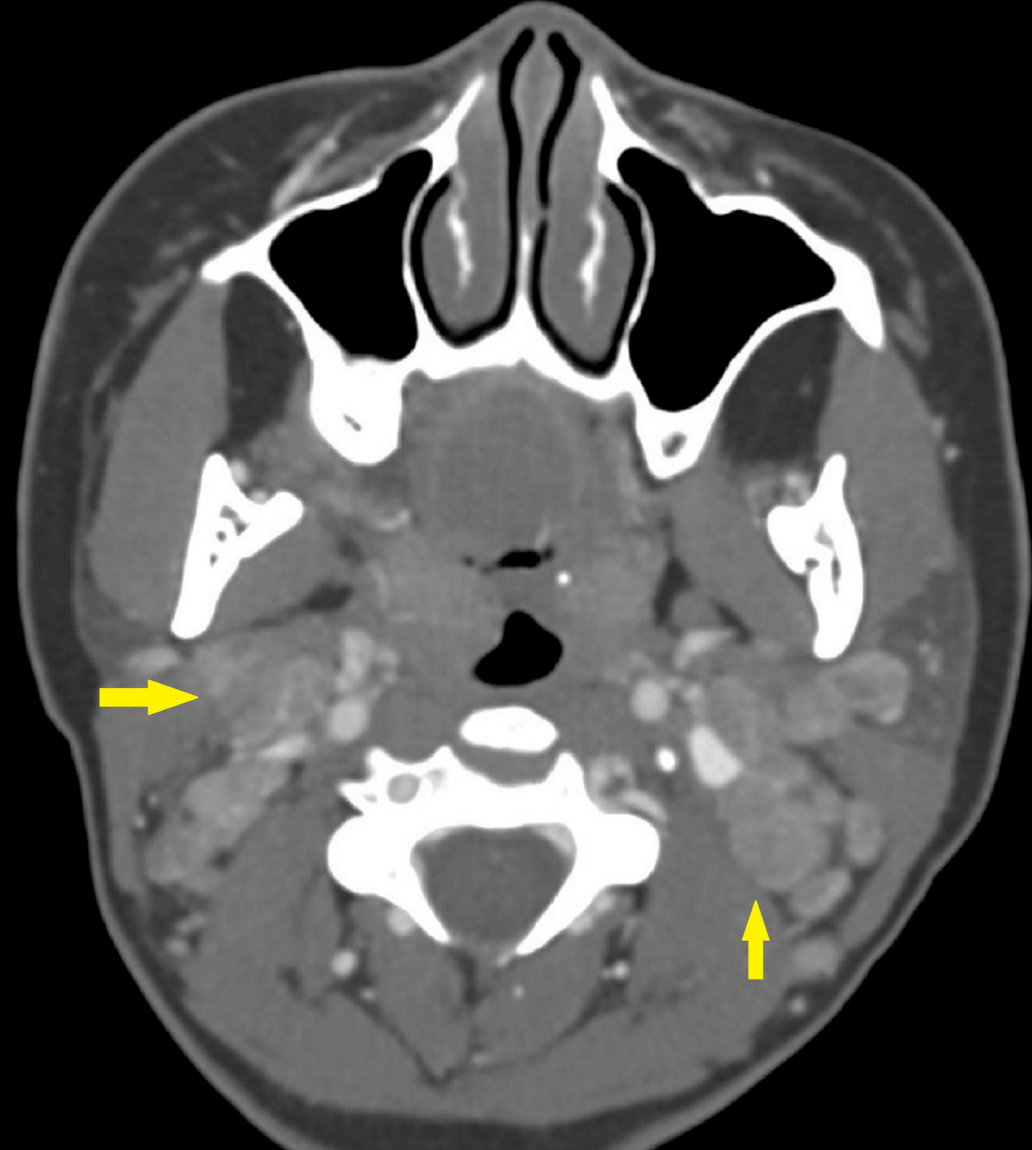
CT neck with contrast, axial view Bilateral enlarged cervical nodes are noted (yellow arrows).

Subsequently, the patient underwent a CT scan of the neck with contrast, which revealed multiple necrotizing axillary and cervical lymphadenopathies bilaterally (Figure 1).

**Figure 2:**
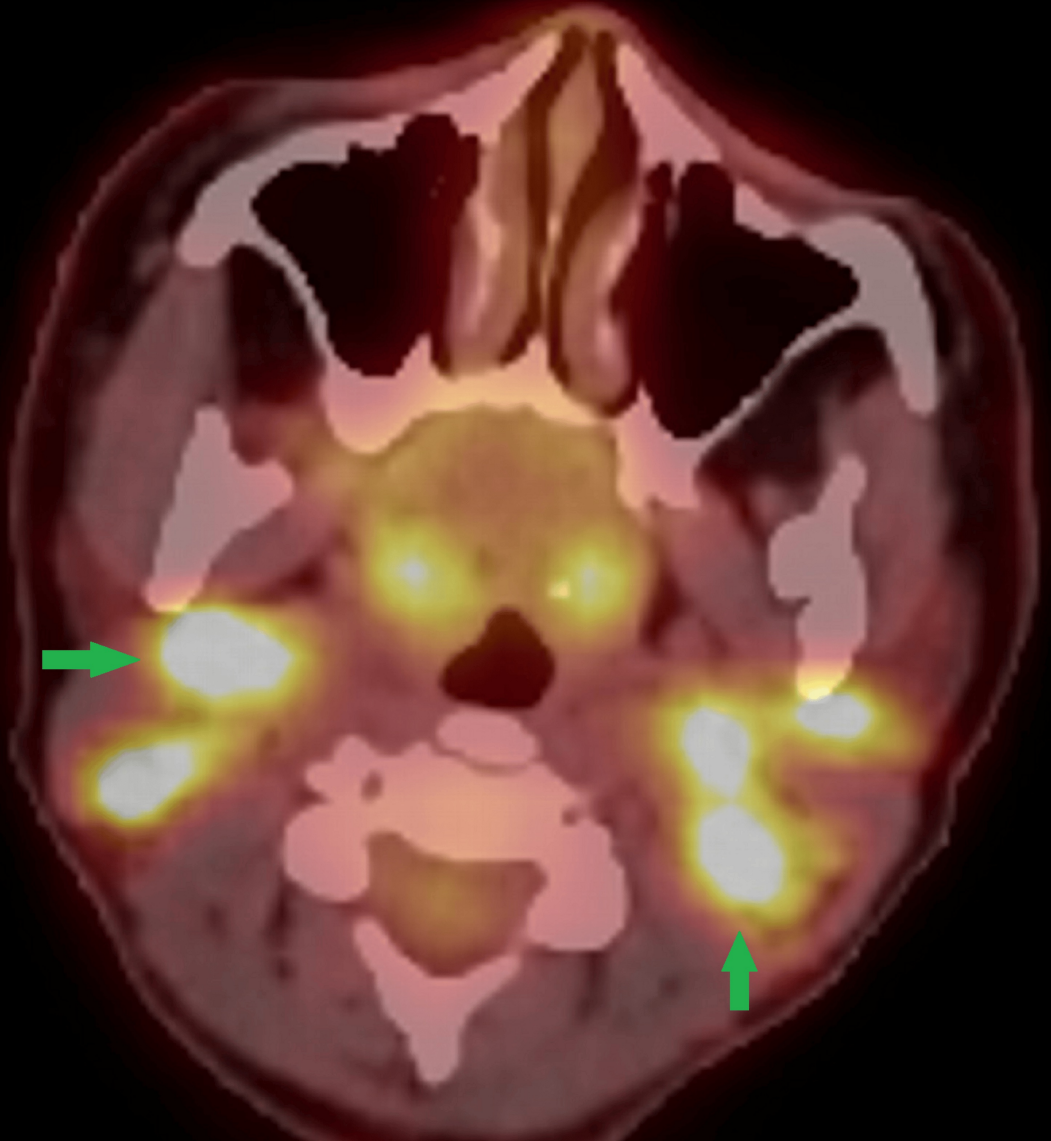
F-18 fluorodeoxyglucose (FDG) PET/CT scan axial fused image Multiple hypermetabolic bilateral cervical nodes are noted (green arrows).

For further evaluation to rule out lymphoma or an occult malignancy, the patient underwent an F-18-fluorodeoxyglucose (FDG) PET/CT scan, which demonstrated multiple enlarged, hypermetabolic cervical, axillary, left supraclavicular, and splenic hilar nodes (Figure 2).

**Figure 3:**
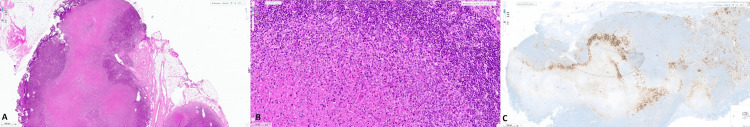
Histopathology of a sampled axillary lymph node (A) 2x magnification of lymph node: necrosis with apoptotic debris; (B) 20x magnification of lymph node: necrosis with apoptotic debris; (C) 2x magnification of lymph node with CD123 staining: necrotic area is surrounded by a rim of pDCs.

The case was then discussed in a multidisciplinary meeting, which advised performing a biopsy of the hypermetabolic nodes. Subsequently, an excision biopsy of an enlarged hypermetabolic axillary node was performed. Histopathological analysis of the node showed multifocal areas of necrosis with apoptotic debris. There were no granulomas, and almost no granulocytes or plasma cells, but the necrotic areas were surrounded by a rim of plasmacytoid dendritic cells (pDCs), CD123, and histiocytes (CD68). The surrounding lymphocytes of the necrotic area were predominantly B lymphocytes (CD20) with scattered immunoblastic cells (CD30). Many interdigitating dendritic cells (S100) were also present. No abnormal proportion existed between B lymphocytes and T lymphocytes (CD20/CD3) (Figure 3).

No microorganisms were detected on histological examination. Additionally, a skin biopsy from a plaque on the left thigh revealed histological features consistent with lupus erythematosus, which are apoptotic keratinocytes, basal vacuolar degeneration, and lymphohistiocytic perivascular and periadnexal inflammatory infiltrate. There was also an increase in pDCs. Furthermore, antinuclear antibody (ANA) testing was performed, which was positive, and there was no renal involvement at the time of evaluation. Based on these radiological and histopathological findings, the patient was diagnosed with coexisting KFD and SLE. Additional viral serologies were also conducted for rubella, mumps, varicella-zoster virus (VZV), cytomegalovirus (CMV), Epstein-Barr virus (EBV), hepatitis A, and hepatitis B due to their possible association with KFD. Results of all these serologies were negative or indicated past exposure only, without active infection, except for hepatitis A, which was positive, reflecting recent or acute infection.

## Discussion

KFD, also known as histiocytic necrotizing lymphadenitis, is a rare cause of cervical lymphadenopathy that predominantly affects women under 30 years of age [3]. While its clinical course often includes nonspecific symptoms such as fever, lymphadenopathy, and cutaneous eruptions, its rarity and overlap with other conditions pose diagnostic challenges [5].

The etiology of KFD remains enigmatic, with various hypotheses postulating both infectious and autoimmune mechanisms [8]. Viral pathogens such as EBV, herpes simplex virus-1 (HSV-1), human papillomavirus (HPV), hepatitis B virus, and parvovirus B19 have been proposed as potential triggers based on their association with an upper respiratory prodrome, atypical lymphocytosis, and the lack of a neutrophilic response [7,8]. However, no conclusive connection has been established [7].

KFD often presents with vague, nonspecific clinical symptoms, making the initial diagnosis challenging [7]. Common presenting signs include fever, chills, sweating, tender lymph nodes, malaise, weight loss, and cough [4,7]. The most prevalent clinical characteristic is unilateral, painful cervical lymphadenopathy, especially in the jugular and posterior cervical nodes [1]. Fever is reported in approximately 35% of cases, along with fatigue, arthralgia, erythematous rash, and, less commonly, hepatosplenomegaly, night sweats, or further weight loss [7]. The lymph node size ranges from 0.5 to 4 cm in most cases, with occasional enlargement up to 6 cm [5]. The lymph nodes are typically firm, mobile, and non-suppurative, although mild tenderness may be present. Generalized lymphadenopathy, involving mediastinal, axillary, retroperitoneal, or inguinal regions, occurs in only 1-22% of cases [4]. While rare, extranodal involvement has been documented in various sites, including the myocardium, bone marrow, salivary glands, uvea, and thyroid [4]. In such cases, systemic symptoms such as fatigue, night sweats, and fever tend to be more pronounced. Laboratory findings are also typically nonspecific, but leukopenia-often neutropenia-being the most consistent abnormality [1,3]. Other lab anomalies may include anemia, thrombocytopenia, elevated C-reactive protein (CRP) and erythrocyte sedimentation rate (ESR), impaired liver function, and atypical lymphocytes on peripheral blood smear [1,7].

The overlap between KFD and other conditions, such as tuberculosis, lymphoma, and SLE, makes the diagnosis particularly complex [1]. The differentiation between lupus lymphadenitis and KFD can be particularly challenging, as both conditions exhibit overlapping clinical features and histological findings [1]. Fine needle aspiration, followed by excisional lymph node biopsy, remains essential for diagnosis, with histopathology often serving as a key role in excluding malignancy and other serious conditions [7]. In 5-30% of KFD cases, cutaneous manifestations can further complicate differentiation from autoimmune conditions such as SLE. These include malar rash, maculopapular lesions, mucosal ulcerations, and lupus-like skin findings. Such features, alongside systemic symptoms, highlight the importance of meticulous histopathological examination and multidisciplinary collaboration in managing complex cases [4].

Given the absence of a specific diagnostic tool for KFD, radiographic examination is essential in the diagnostic process [3]. In patients with cervical lymphadenopathy, CT and MRI are considered valuable modalities, with sonography also utilized in certain instances. Ultrasonography usually demonstrates lymph nodes with a hypoechoic center and a hyperechoic rim, findings that may also suggest necrotic lymphadenopathy [9]. CT findings in KFD often reveal multiple, mildly enlarged lymph nodes (0.5-3.5 cm, with a mean size of 1.62 cm), which are generally homogenous but can sometimes exhibit nodal necrosis. Perinodal infiltration, a hallmark of inflammatory diseases, is frequently observed and corresponds to histologic infiltration of lymphoid cells, histiocytes, and karyorrhectic debris [3]. Although nodal necrosis is less common, it is not unusual and can mimic other conditions such as lymphoma, metastases, or tuberculosis. These features highlight the diagnostic challenge of distinguishing KFD from malignant or infectious conditions, particularly when necrosis is evident [9].

As the patient was admitted to the hospital and received improper treatment before a proper diagnosis was made, this case serves as an example of the diagnostic difficulties associated with KFD. The combination of systemic symptoms and cervical node involvement in our patient highlighted the case's diagnostic difficulty. The CT findings of multiple necrotizing lymphadenopathies in the bilateral cervical, axillary, and splenic hilar lymphadenopathies, which were hypermetabolic on the corresponding PET-CT scan, further reinforced by the histopathological findings, played a crucial role in distinguishing KFD from other causes of lymphadenitis in our case.

The differential diagnosis of a slow-growing neck mass encompasses a wide range of entities, including malignant lymphoma, SLE, Hodgkin disease, toxoplasmosis, metastatic carcinoma, infectious mononucleosis, acquired immunodeficiency syndrome, cat-scratch disease, and angioimmunoblastic lymphadenopathy [5,9]. A key distinction between KFD and lymphoma lies in the size and distribution of lymphadenopathy. KFD typically manifests with multiple, mildly enlarged lymph nodes, often smaller than 3 cm in diameter, whereas lymphoma is characterized by fewer, moderately to markedly enlarged lymph nodes [9]. These features and histopathological findings are pivotal in distinguishing KFD from other diagnoses.

## Conclusions

KFD is a rare, self-limiting, and benign disease with a good prognosis when promptly diagnosed and managed appropriately. Contrary to the prevalent literature, KFD does not always precede SLE. Occasionally, they coexist, making early diagnosis challenging.

Clinicians' expertise in diagnosing conditions, particularly when evaluating patients with fever and lymphadenopathy, is paramount. Many patients with these symptoms are erroneously diagnosed with lymphoma or, occasionally, tuberculosis, which can lead to ineffective treatment.
